# Effects of *APOE* Genotype and Western Diet on Metabolic Phenotypes in Female Mice

**DOI:** 10.3390/metabo13020287

**Published:** 2023-02-16

**Authors:** Amy Christensen, Christian J. Pike

**Affiliations:** Davis School of Gerontology, University of Southern California, Los Angeles, CA 90089, USA

**Keywords:** apolipoprotein E, behavior, inflammation, metabolic stress, obesogenic diet

## Abstract

Western diets high in sugars and saturated fats have been reported to induce metabolic and inflammatory impairments that are associated with several age-related disorders, including Alzheimer’s disease (AD) and type 2 diabetes (T2D). The apolipoprotein E (*APOE*) genotype is associated with metabolic and inflammatory outcomes that contribute to risks for AD and T2D, with the *APOE4* genotype increasing risks relative to the more common *APOE3* allele. In this study, we investigated the impacts of the *APOE* genotype on systemic and neural effects of the Western diet. Female mice with knock-in of human *APOE3* or *APOE4* were exposed to control or Western diet for 13 weeks. In the control diet, we observed that *APOE4* mice presented with impaired metabolic phenotypes, exhibiting greater adiposity, higher plasma leptin and insulin levels, and poorer glucose clearance than *APOE3* mice. Behaviorally, *APOE4* mice exhibited worse performance in a hippocampal-dependent learning task. In visceral adipose tissue, *APOE4* mice exhibited generally higher expression levels of macrophage- and inflammation-related genes. The cerebral cortex showed a similar pattern, with higher expression of macrophage- and inflammation-related genes in *APOE4* than *APOE3* mice. Exposure to the Western diet yielded modest, statistically non-significant effects on most metabolic, behavioral, and gene expression measures in both *APOE* genotypes. Interestingly, the Western diet resulted in reduced gene expression of a few macrophage markers, specifically in *APOE4* mice. The observed relative resistance to the Western diet suggests protective roles of both female sex and young adult age. Further, the data demonstrate that *APOE4* is associated with deleterious systemic and neural phenotypes and an altered response to a metabolic stressor, findings relevant to the understanding of interactions between the *APOE* genotype and risks for metabolic disorders.

## 1. Introduction

Apolipoprotein E (ApoE) is a cholesterol transport protein that is coded for by three different alleles of the *APOE* gene, *APOE2*, *APOE3*, and *APOE4*. These *APOE* alleles generate ApoE proteins that vary at amino acids 112 and 158 and show significant functional differences [1]. Importantly, *APOE* genotype impacts well established associations among Alzheimer’s disease (AD), type 2 diabetes (T2D), and cardiovascular disease (CVD) [2]. *APOE3* is the most common allele in human populations and is used as the reference for establishing relative risk. In comparison to *APOE3*, *APOE4* increases risk as much as 15-fold for AD [3,4,5] and two- to three-fold for T2D and CVD [6]. Conversely, *APOE2* is associated with decreased risks for AD [7,8] and CVD [9]. Interestingly, the *APOE* genotype shows parallel relationships with lifespan in both humans and mice. *APOE2* is associated with increased longevity, and *APOE4* is associated with decreased longevity [10,11]. The mechanism(s) by which *APOE* affects aging and age-related disease risks is unclear, but ApoE modulates aspects of metabolism [12], lipid dynamics, and inflammation, all of which are also implicated in pathways relevant to these age-related diseases.

Obesity is an important factor that links *APOE*, AD, CVD, and T2D. Risks for AD, CVD, and T2D are all strongly increased by obesity [13,14,15], interactions that can be impacted by the *APOE* genotype. For example, abundant evidence indicates that relationships between obesity and AD risk can be worsened by *APOE4* [16,17]. Both *APOE4* and obesity affect similar pathways associated with metabolic stress, including inflammation. Many prior studies have shown higher markers of inflammation in *APOE4* carriers [18,19,20,21], although the relationship between the *APOE* genotype and inflammatory tone appears to be complex and may depend upon context [22,23,24]. Inflammatory pathways are implicated in pathological and normal age-related cognitive decline [25,26,27] and represent one mechanism by which obesity and *APOE* may interact to affect vulnerability to AD and other age-related metabolic disorders. 

In the present study, we investigated the effects of the *APOE* genotype on systemic and neural responses to an obesogenic diet. As *APOE4*-associated cognitive [28,29] and metabolic [30] risks are more robust in females, we studied female mice. In prior work with AD transgenic mice with knock-in of human *APOE3* or *APOE4*, we observed that young adult female *APOE3* mice showed more metabolic dysfunction and greater AD-related pathology and behavioral impairment in response to an obesogenic diet than *APOE4* females [31]. However, because even early AD-related pathology can result in systemic metabolic impairments [32], understanding the metabolic consequences of *APOE* genotype requires analyses in the absence of AD transgenes. This was accomplished by assessing metabolic and cognitive responses in *APOE* knock-in mice on a 13-week exposure to control versus Western diet that is high in both sugars and saturated fats [28,29,30]. Our findings provide new insights into the effects of *APOE* genotype on metabolic stressors associated with AD risk.

## 2. Methods

### 2.1. Animals

A colony of EFAD mice [33] were maintained at vivarium facilities at the University of Southern California from breeder mice generously provided by Dr. Mary Jo LaDu (University of Illinois at Chicago). EFAD mice are heterozygous for 5xFAD transgenes and homozygous for knock-in of human *APOE3* or *APOE4*. Breeding strategies for EFAD mice yield litters that are ~50% 5xFAD^+/−^ (EFAD) and ~50% 5xFAD^−/−^ (EFAD-non-carriers, EFAD-NC). The latter are homozygous for *APOE,* but they lack AD-related transgenes. In this study, female EFAD-NC mice homozygous for *APOE3* (*APOE3* mice) or *APOE4* (*APOE4* mice) were maintained under controlled temperature, a 12:12 light/dark schedule (lights on at 06:00), and with ad libitum access to food and water. At 2.5 months of age, mice were randomly assigned (*n* = 7–8/group) to either ingredient-matched control (10% calories from fat and 7% from sugar; catalog #D12450J, Research Diets, Inc., New Brunswick, NJ, USA) or Western (WD; 45% calories from fat and 17% from sugar; catalog #D12451, Research Diets, Inc.) diets. Body weight was monitored weekly for the 13-week diet exposure period. All procedures were conducted under a protocol approved by the USC Institution for Animal Care and Use Committee and under the supervision of USC veterinarians. 

### 2.2. Glucose Tolerance Test

A glucose tolerance test was performed 12 weeks after the start of the diets. Animals were fasted overnight (~16 h) and orally gavaged with 2 g/kg D-glucose. Blood glucose levels were measured at 0, 15-, 30-, 60-, and 120-min following glucose administration. Five microliters of blood were collected on a glucose test strip and assayed using a Precision Xtra glucose monitor (Abbott Laboratories, Abbott Park, IL, USA).

### 2.3. Tissue Collection

At the end of the 13-week treatment period, *APOE3* and *APOE4* mice were euthanized by carbon dioxide overdose, following overnight fasting, after which the brain was rapidly removed, and the cerebral cortex was dissected and stored as frozen. Blood was collected and kept on ice prior to centrifugation to collect plasma, which was stored in aliquots at −80° until assayed. The retroperitoneal and visceral (including gonadal and uterine fat) fat pads were dissected, weighed, and frozen.

### 2.4. Insulin, Leptin, and Cholesterol Assays

Fasting levels of insulin and leptin were determined using rat/mouse insulin (Millipore, Catalog #EZRMI-13K) and mouse leptin (Millipore, Catalog # EZML-82K) ELISA kits, according to the manufacturers’ instructions, using plasma collected at euthanization. Plasma cholesterol was measured using a colorimetric cholesterol quantitation assay kit (Abcam, Catalog #ab65359), according to the manufacturer’s directions. Two *APOE3* control and two *APOE3* WD animals exhibited negative insulin concentrations and were excluded from insulin analyses. One *APOE3* control animal exhibited a negative leptin concentration and was excluded from leptin analysis.

### 2.5. Behavior

Mice were behaviorally assessed at week 11 using object placement (NOP) and novel object recognition (NOR), tasks that assess cognition and learning and memory involving hippocampal and parahippocampal regions [34]. Mice were habituated to the testing arena without objects for 5 min per day for three days prior to testing. Before testing each day, animals were habituated to the room for at least 30 min. On the test day, two identical objects were placed into the box, and the mice were allowed to explore for five minutes (training). Twenty minutes later, the animals were placed again in the chamber with the same objects, but one moved 90° in the chamber (NOP) and was allowed to explore for five minutes. Twenty minutes after NOP, the mice were placed in the chamber with one familiar object and one novel object for five minutes (NOR). The time the mice spent exploring the objects was recorded. The discrimination index was calculated as (time with novel—time with familiar)/total time with objects. The recognition index was calculated as time with novel/total time with objects. Mice were excluded from analysis if they spent less than 6 s total exploring the objects. In total, two mice were excluded from NOP, and four mice were excluded from NOR.

### 2.6. Quantitative Real-Time PCR

RNA was extracted from visceral adipose tissue or the cerebral cortex by lysis with TRIzol reagent (Life Technologies, Carlsbad, CA, USA, Cat #15596018), according to the manufacturer’s protocol, except for in adipose tissue. For adipose tissue, tissue was lysed using a dounce homogenizer for 20 strokes. The solution was centrifuged for 10 min, and the top layer (fat) was removed prior to the addition of chloroform. After the completion of the TRI-zol protocol, potential genomic DNA contamination was removed from all samples by an RNase-free DNase treatment (Lucigen, Middlesex, UK, Cat #D9905K). Purified RNA (1 μg) was used for reverse transcription using the iScript synthesis system (Bio-Rad, Hercules, CA, USA, Cat #1708891). The resulting cDNA was used for quantitative PCR using a Bio-Rad CFX96 Touch Real-Time PCR Detection System. Standard PCR cycling protocols were used, consisting of 40 cycles, with denaturation at 95 °C and annealing at 60 °C. The amplification efficiency was estimated from the standard curve for each gene. All primers have an efficiency of 88–115%. Relative quantification of mRNA levels was determined by the ΔΔCt method. All groups were compared to the *APOE3* control. All experimental primers were compared to the expression of β-actin in the brain and the average expression of succinate dehydrogenase complex, subunit A (SDHA), and hypoxanthine guanine phosphoribosyltransferase (HPRT) in adipose tissue. The expression of control primers showed no significant differences according to the *APOE* genotype or diet (2-way ANOVA). Primer sequences (Life Technologies) are listed in Table 1.

### 2.7. Statistics

All data are reported as means + standard errors of the mean. Raw data were analyzed using GraphPad Prism 8. Most data were analyzed using two-way ANOVAs (*APOE* × diet), with Tukey’s *post hoc* tests where applicable. Changes in body weight and glucose tolerance over time were analyzed using three-way repeated measure ANOVAs (*APOE* × diet × time). Initial body weights were compared by *t*-test. Comparisons with *p* < 0.05 were considered statistically significant. Because several comparisons yielded *p* values only slightly above the significance limit, outcomes with *p* ≤ 0.10 are also noted. All relevant statistics are listed in Table 2 and noted in the figures.

## 3. Results

### 3.1. Metabolic Consequences of APOE Genotype and Western Diet

**Figure 1:**
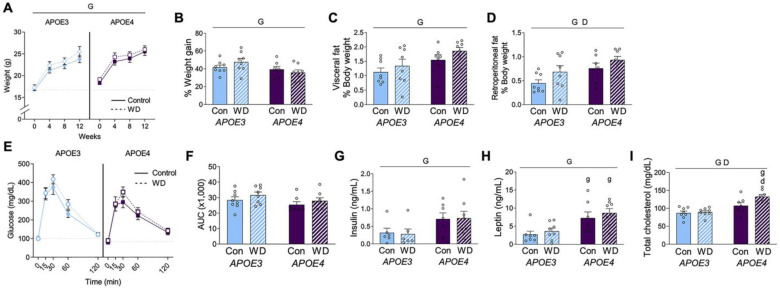
Metabolic profiles in *APOE* mice after Western diet. (**A**) Body weight was measured at the start and at four-week intervals in female *APOE* mice on control (Con; open circles) and Western diet (WD; filled squares). The dotted line indicates the initial weight of the *APOE3* mice. (**B**) The percent weight change was calculated from the beginning to the end of the experimental period. (**C**,**D**) Fat pads from (**C**) visceral and (**D**) retroperitoneal depots were removed and weighed at the conclusion of the experiment. Values are expressed as a percentage of body weight. (**E**) Results from an oral glucose tolerance test that was administered after 12 weeks of diet. (**F**) The area under the curve was calculated for the GTT. (**G**–**I**) Plasma was collected under fasting conditions at the end of the experiment and measured for (**G**) insulin, (**H**) leptin, and (**I**) cholesterol. Data are shown as individual values (open circles) and mean values (+SEM) from *n* = 7–8 mice per group. “G” indicates a significant main effect of the *APOE* genotype, “D” indicates a significant main effect of diet, “g” indicates a significant difference from the *APOE3* mice on the same diet, “d” indicates a significant difference from animals with the same *APOE* genotype on the control diet. Significance is *p* < 0.05.

Female mice were maintained on control or Western diet (WD) for 13 weeks. At the start of the treatment period, *APOE4* mice had significantly heavier body weight than *APOE3* mice (Figure 1A; *t*-test, *p* < 0.001). There was a significant effect of *APOE* genotype on body weight over the course of the study (see Table 2 for statistical analyses). By the end of the 13-week treatment period, *APOE3* and *APOE4* mice were of similar weight. Body weight increased across all groups regardless of diet, although the percent weight change was significantly greater in *APOE3* mice compared to *APOE4* mice (Figure 1B). There was a significant main effect of genotype on adiposity, with *APOE4* mice exhibiting significantly larger visceral and retroperitoneal fat pads (normalized to body weight; Figure 1C,D, respectively). Diet did not significantly affect body weight or visceral fat mass, but it was associated with increased retroperitoneal fat.

An oral glucose tolerance test was administered 12 weeks after the start of diet (Figure 1E). The area under the curve appeared to be greater in *APOE3* mice, but this was not statistically significant (Figure 1F). Terminal levels of plasma insulin and leptin were significantly higher in *APOE4* females (Figure 1G,H), with no significant effect of diet in either genotype. Plasma cholesterol was significantly increased in *APOE4* females and by WD, though this diet increase was driven by a change in the *APOE4* mice (Figure 1I).

### 3.2. Cognitive Effects of APOE Genotype and Western Diet in APOE Mice on the Western Diet

**Figure 2:**
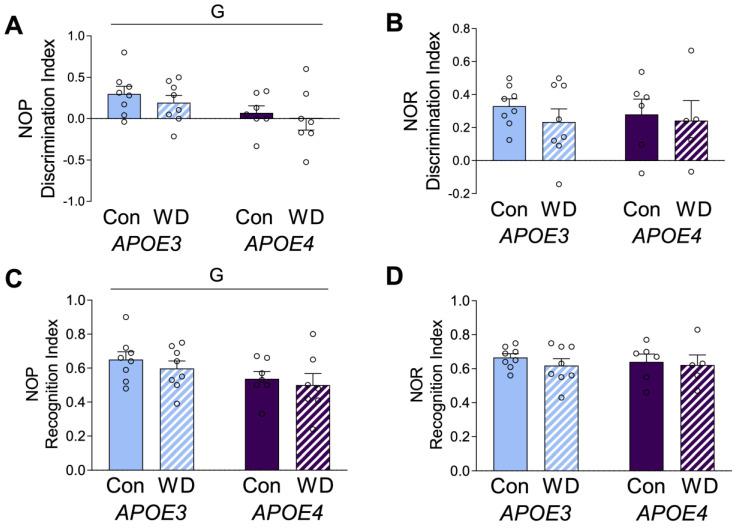
Behavioral effects in *APOE* mice after the Western diet. Animals were tested with (**A**,**C**) novel object placement (NOP) and (**B**,**D**) novel object recognition (NOR) behavioral tasks. The discrimination index is represented in (**A**,**B**), with values > 0 indicating positive performance. The recognition index is represented in (**C**,**D**). Data are shown as individual values (open circles) and mean values (+SEM), from *n* = 5–8 mice per group. “G” indicates a significant main effect of the *APOE* genotype. Significance is *p* < 0.05.

*APOE* mice were tested on the novel object placement and novel object recognition tests. Novel object placement primarily evaluates spatial learning and relies mostly on hippocampal learning. Novel object recognition relies on non-spatial learning and engages multiple brain regions [35]. *APOE4* animals showed significantly poorer performance in novel object placement (Figure 2A,C), but there was no genotype difference in performance in novel object recognition (Figure 2B,D). There were no significant effects of diet on either behavioral measure, which are presented as both discrimination index (Figure 2A,B) and recognition index (Figure 2C,D).

### 3.3. Adipose Gene Expression with the APOE Genotype and the Western Diet

**Figure 3:**
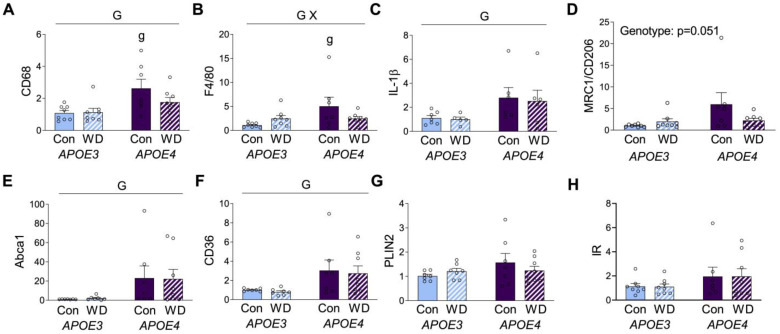
Gene expression levels in visceral adipose tissue. Results of mRNA quantification studies in *APOE3* (blue) and *APOE4* (purple) mice maintained on control (Con; solid bars) or Western (WD; hatched bars) diets. Gene targets included the macrophage markers (**A**) CD68 and (**B**) F4/80, the inflammation-related markers (**C**) IL-1β and (**D**) CD206, and the metabolic macrophage markers (**E**) Abca1, (**F**) CD36, (**G**) PLIN2, and (**H**) insulin receptor (IR). Data show fold difference as individual values (open circles) and means (+SEM) relative to the Con *APOE3* group from *n* = 7–8 mice per group. “G” indicates a significant main effect of *APOE* genotype, “X” indicates a significant interaction effect between *APOE* and diet, and “g” indicates a significant difference from *APOE3* mice on the same diet. Significance is *p* < 0.05.

As both obesogenic diets and the *APOE* genotype are known to affect adiposity, we examined mRNA expression levels of several genes in visceral adipose tissue. We first assessed two well-established macrophage markers, CD68 and F4/80. There was a main effect of genotype, such that expression of both was higher in *APOE4* mice (Figure 3A,B). Interestingly, *post hoc* analyses showed significant *APOE3* vs. *APOE4* differences in CD68 and F4/80 expression only under the control diet. Although there was no significant main effect of diet on CD68 and F4/80 expression, there was a significant interaction for F4/80 expression, wherein WD was associated with increased levels in *APOE3,* but lower levels in *APOE4* relative to the control diet (Figure 3B). CD68 showed a similar trend of lower expression, with WD only in *APOE4* mice (Figure 3A).

Macrophages have traditionally been separated into M1 and M2 phenotypes, which are characterized by predominantly pro- or anti-inflammatory properties, respectively. Although an oversimplification, these categories can provide insights into macrophage biology. The M1 marker interleukin-1β (IL-1β) was significantly increased in *APOE4* mice, regardless of diet (Figure 3C). Expression of the M2 marker mannose receptor C type 1 or cluster of differentiation 206 (MRC or CD206) showed a pattern similar to CD68 and F4/80, with a nonsignificant trend (*p* = 0.056) of higher levels in *APOE4* mice under control diet, but not for those of WD (Figure 3D). Macrophages can also exhibit metabolic phenotypes that are associated with obesity. The main markers of these metabolic macrophages are ATP binding cassette subfamily A type 1 (ABCA1), cluster of differentiation 36 (CD36), and perilipin 2 (PLIN2) [36], although these markers are not exclusive to macrophages and can also be expressed by adipocytes. *APOE4* mice showed higher expression of both ABCA1 and CD36, with no significant modulatory effect of diet (Figure 3E,F). PLIN2 expression did not significantly differ across *APOE* or diet (Figure 3G). Further, given the observed differences in insulin and glucose levels, we probed for insulin receptor (IR) expression in the adipose tissue, but we saw no significant differences (Figure 3H).

### 3.4. Cortex Gene Expression with APOE Genotype and Western Diet

**Figure 4:**
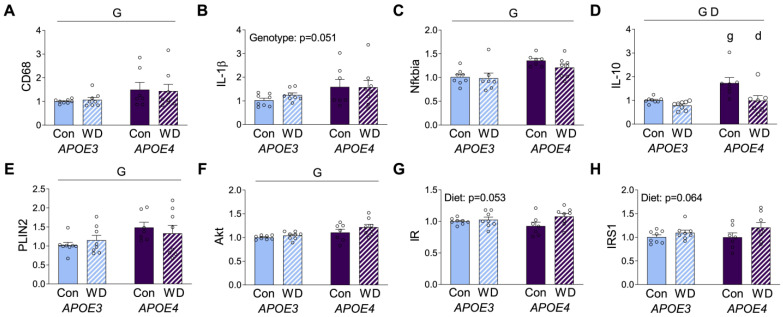
RNA expression levels in cerebrocortical brain tissue. Results of mRNA quantification studies in *APOE3* (blue) and *APOE4* (purple) mice maintained on control (Con; solid bars) or Western (WD; hatched bars) diets. Gene targets included the macrophage marker, (**A**) CD68, the inflammatory markers (**B**) IL-1β, (**C**) Nfkbia, and (**D**) IL-10, and the metabolic markers (**E**) PLIN2, (**F**) Akt, (**G**) IR, and (**H**) IRS1. Data show fold difference as individual values (open circles) and mean values (+SEM) relative to the Con *APOE3* group from *n* = 7–8 mice per group. “G” indicates a significant main effect of *APOE* genotype, “D” indicates a significant main effect of diet, “g” indicates a significant difference from *APOE3* mice on the same diet, and “d” indicates a significant difference from animals with the same *APOE* genotype on the control diet. Significance is *p* < 0.05.

In order to investigate diet and *APOE* genotype interactions in the brain, mRNA expression of selected inflammatory and metabolic genes was probed in the cerebral cortex. The macrophage marker CD68 showed higher levels in *APOE4* mice, with no significant effect of diet (Figure 4A). The pro-inflammatory cytokine IL-1β exhibited a similar pattern of increased expression in *APOE4* mice, although it did not meet the significance criterion (*p* = 0.051, Figure 4B). Likewise, the inflammatory regulator NFκB inhibitor alpha (NFκBIA) that modulates NFκB activity was also significantly higher in *APOE4* mice, but it was not significantly affected by WD in either genotype (Figure 4C). The anti-inflammatory cytokine interleukin-10 (IL-10) showed significant effects regarding both *APOE* genotype and diet, with *APOE4* increasing and WD decreasing expression levels (Figure 4D). 

Upon assessing metabolic markers, we first examined the metabolic macrophage markers, ABCA1 and CD36, and we found that they were not affected by *APOE* or diet (data not shown). However, PLIN2 was increased in the cortex of *APOE4* relative to *APOE3,* but it was not affected by WD (Figure 4E), confirming recent work showing greater expression of this protein in *APOE4* astrocytes [37]. We next probed expression of the insulin related genes AKT, insulin receptor (IR), and insulin receptor substrate-1 (IRS-1). AKT expression showed modest, but significantly higher levels, in *APOE4* mice with no effect of diet (Figure 4F). In contrast, both IR and IRS-1 showed no *APOE* genotype differences and nonsignificant trends (*p* = 0.053 and *p* = 0.064, respectively) towards increased expression with WD (Figure 4G,H).

## 4. Discussion

Peripheral and neural effects of the *APOE* genotype and the Western diet were explored in young adult female mice. Interestingly, there were few effects of diet in either the *APOE3* or *APOE4* mice, although *APOE4* mice were relatively impaired regarding most measures. *APOE4* mice had larger fat pads and worse metabolic and cognitive phenotypes. In addition, *APOE4* mice generally exhibited higher expression of macrophage and inflammation-related markers in adipose and brain tissue. For a subset of these markers, the Western diet was associated with reduced expression, specifically within *APOE4* mice. Note that these findings are limited to gene expression and may not fully reflect changes detectable by histological analyses.

The generally modest effects of Western diet in female mice observed in this study are not altogether surprising. Young females have been shown previously to be resistant to many of the effects of high-fat diets [38,39,40]. Indeed, in both wild type [41] and AD transgenic [42] mice, females have been reported to exhibit little metabolic response to obesogenic diets. This effect appears to be age-dependent, such that, by middle age, the sex difference partially reverses, yielding greater diet-induced effects in females than in males [43]. These sex differences may be due, in part, to the effects of estrogens, which are much higher in young adult females, but they decline in abundance and or efficacy with increasing age. Estrogens have effects on both the brain and peripheral tissues implicated in protecting females from many of the deleterious effects of obesogenic diets [44,45,46]. 

Prior studies of obesogenic diets in *APOE* knock-in mice suggest both *APOE* genotype and sex differences are relevant predictors of vulnerability. Perhaps the most consistent finding has been greater weight gain in *APOE3* mice [47,48], which aligns with our observation that *APOE3* females on both diets showed proportionally larger increases in body weight. Note, however, that *APOE4* mice had a significantly higher body weight at the start of the study, indicating complexity in the effects of the *APOE* genotype and body weight. The literature on *APOE*-dependent metabolic differences is somewhat mixed, with evidence of both similar outcomes in *APOE3* vs. *APOE4* mice [49,50] and greater metabolic impairments in *APOE4* mice [51,52]. Although we observed only modest metabolic effects of the Western diet, our results support prior observations of metabolic impairments in *APOE4* mice. In one study that compared both sexes in adult, but not aged *APOE* mice, male mice showed stronger diet-induced metabolic responses, with greater susceptibility in *APOE4* mice, whereas females showed similar metabolic responses across *APOE* genotypes [49]; our results align with the latter finding. Interestingly, estrogen status may be important for not only the metabolic resistance of females, but perhaps also for masking *APOE4* vulnerability. In a recent study, an experimental model of menopause/ovarian failure, combined with high-fat diet, worsened cognitive outcomes more strongly in *APOE4* females [53]. There have also been reports of obesogenic diets in *APOE* knock-in mice crossed into AD transgenic models. In the EFAD mouse model, the Western diet yielded similar or greater metabolic impairments in *APOE3* mice of both sexes, but it increased AD-related pathology only in *APOE3* for females [31] and *APOE4* for males [54]. In APP/PS1ΔE9 mice with human *APOE*, the obesogenic diet increased pathology only in the *APOE4* genotype, and this had a stronger effect in males [55]. Collectively, our results complement an increase in the literature that is defining the modulatory roles of sex and *APOE* genotype on their effects on metabolic stress that is linked to disease risk.

Particularly interesting are our findings on the mRNA expression of macrophage- and inflammation-related genes. We observed that levels of these markers were generally higher in adipose tissue and cerebral cortex of *APOE4* mice, a finding consistent with prior findings, indicating an exaggerated inflammatory tone with the *APOE4* genotype [56,57]. Perhaps unexpected was the observed general trend of lower adipose levels of macrophage and inflammation markers with Western diet, specifically in *APOE4* mice. The cerebral cortex showed limited diet effects; the hippocampus was not examined, but prior work indicates similar effects of obesogenic diet on the cortex and the hippocampus [58,59]. Consistent with our results, lower levels of inflammation-related factors in the liver and the plasma in *APOE4* relative to *APOE3* mice following the Western diet have been reported [23]. In a recent study, cultured microglia from *APOE4* mice have been shown to have increased cytokine production and lipid accumulation compared to *APOE3* microglia in the absence of other stressors [60]. However, the obesogenic diet was reported to result in both microglial and astrocytic gliosis in *APOE3,* but not *APOE4,* mice [61]. Further, the obesogenic diet in female *APOE4* mice reduced the microglia/macrophage marker CD68 [62]. Together, these findings may suggest that *APOE4* is associated with an altered inflammation-related response to metabolic stressors. 

In summary, these results extend and confirm previous evidence that *APOE4* yields systemic and neural phenotypes characterized by metabolic and cognitive impairments even in the absence of significant aging or AD-related pathology. Although obesogenic diets are known to induce metabolic and cognitive dysfunctions, the Western diet caused only very modest changes in this study. We suggest that the observed resistance to the obesogenic diet is related to both female sex and young adult age of the *APOE* mice. It is important to interpret the cognitive data with caution, as a relatively small number of mice were tested. Our finding that the Western diet reduces expression of inflammatory markers, specifically in *APOE4* mice, suggests an *APOE4*-dependent alteration in inflammatory responses to metabolic stress, which in turn implicates inflammatory more than metabolic pathways in contributing to the impacts of the *APOE* genotype on the consequences of the obesogenic diet.

## Figures and Tables

**Table 1:** Primer sequences used in PCR analyses.

Gene Target	Forward Primer	Reverse Primer	Amplicon Size
*CD68*	TTCTGCTGTGGAAATGCAAG	AGAGGGGCTGGTAGGTTGAT	241 bp
*F4/80*	TGCATCTAGCAATGGACAGC	GCCTTCTGGATCCATTTGAA	169 bp
*IL-1β*	GGGCCTCAAAGGAAAGAATC	TACCAGTTGGGGAACTCTGC	183 bp
*MRC1/CD206*	ATGCCAAGTGGGAAAATCTG	TGTAGCAGTGGCCTGCATAG	153 bp
*Abca1*	ATATGCGCTATGTCTGGGGC	GCGACAGAGTAGATCCAGGC	205 bp
*CD36*	TATTGGTGCAGTCCTGGCTG	CTGCTGTTCTTTGCCACGTC	201 bp
*PLIN2*	GTTATGGTCTTGCCCCAGCT	ATGAAGCCTGCTCAGACCAC	222 bp
*Nfkbia*	TGCCTGGCCAGTGTAGCAGTCTT	CAAAGTCACCAAGTGCTCCACGAT	149 bp
*IL-10*	CCAGGGAGATCCTTTGATGA	CATTCCCAGAGGAATTGCAT	173 bp
*Akt*	GAGAACCGTGTCCTGCAGAA	GTTCTCCAGCTTCAGGTCCC	261 bp
*IR*	GAGTATGACGACTCGGCCAG	CCTGTGCTCCTCCTGACTTG	252 bp
*IRS1*	AATGTGTGGCTGAGACCTGG	GCTGATGCTGGCATAGTTGC	249 bp
*β-actin*	AGCCATGTACGTAGCCATCC	CTCTCAGCTGTGGTGGTGAA	231 bp
*SDHA*	ACACAGACCTGGTGGAGACC	GGATGGGCTTGGAGTAATCA	156 bp
*HPRT*	AAGCTTGCTGGTGAAAAGGA	TTGCGCTCATCTTAGGCTTT	186 bp

Abbreviations: Cluster of differentiation 68, CD68; EGF-like module-containing mucin like hormone receptor-like 1, F4/80; interleukin-1β, IL-1β; mannose receptor C1, MRC1/CD206; ATP-binding cassette transporter A1, Abca1; cluster of differentiation 36, CD36; perilipin 2, PLIN2; NFκB inhibitor alpha, Nfkbia; interleukin-10, IL-10; insulin receptor, IR; insulin receptor substrate-1, IRS1; succinate dehydrogenase complex, subunit A, SDHA; hypoxanthine guanine phosphoribosyltransferase, HPRT; base pairs, bp.

**Table 2:** Statistical analyses with significant or nearly significant outcomes.

Figure	Main Effects and Interactions	*post-hoc* Tests
1A	*F*_APOE(1,27)_ = 5.2, *p* < 0.05 *F*_time(2.34,63.3)_ = 267, *p* < 0.0001	0 weeks: *APOE3* control vs. *APOE4* WD, *p* < 0.01
1B	*F*_APOE(1,27)_ = 4.7, *p* < 0.05	*APOE3* WD vs. *APOE4* WD, *p* = 0.07
1C	*F*_APOE(1,27)_ = 7.4, *p* < 0.05	
1D	*F*_APOE(1,27)_ = 8.1, *p* < 0.01 *F*_diet(1,27)_ = 4.5, *p* < 0.05	
1E	*F*_APOE(1,27)_ = 4.6, *p* < 0.05 *F*_time(2.65,71.6)_ = 147, *p* < 0.0001 *F*_timeXAPOE(4,108)_ = 3.4, *p* < 0.05	
1F	No significant differences	
1G	*F*_APOE(1,23)_ = 6.1, *p* < 0.05	
1H	*F*_APOE(1,26)_ = 16.5, *p* < 0.001	*APOE3* control vs. *APOE4* control; *p* < 0.05 *APOE3* WD vs. *APOE4* WD; *p* < 0.05
1I	*F*_APOE(1,27)_ = 27.6, *p* < 0.0001 *F*_diet(1,27)_ = 5.1, *p* < 0.05	*APOE3* WD vs. *APOE4* WD; *p* < 0.0001 *APOE4* Control vs. *APOE4* WD; *p* < 0.05
2A	*F*_APOE(1,26)_ = 4.4, *p* < 0.05	
2B	No significant differences	
2C	*F*_APOE(1,26)_ = 4.3, *p* < 0.05	
2D	No significant differences	
3A	*F*_APOE(1,27)_ = 10.4, *p* < 0.01	*APOE3* control vs. *APOE4* control; *p* < 0.05
3B	*F*_APOE(1,27)_ = 4.4, *p* < 0.05 *F*_APOEXdiet_(_1,27)_ = 4.4, *p* < 0.05	*APOE3* control vs. *APOE4* control; *p* < 0.05
3C	*F*_APOE(1,20)_ = 6.3, *p* < 0.05	
3D	*F*_APOE(1,27)_ = 4.2, *p* = 0.051	
3E	*F*_APOE(1,25)_ = 6.5, *p* < 0.05	
3F	*F*_APOE(1,25)_ = 8.4, *p* < 0.01	
3G	No significant differences	
3H	No significant differences	
4A	*F*_APOE(1,27)_ = 4.4, *p* < 0.05	
4B	*F*_APOE(1,27)_ = 4.2, *p* = 0.051	
4C	*F*_APOE(1,26)_ = 14.9, *p* < 0.001	
4D	*F*_APOE(1,26)_ = 9.4, *p* < 0.01 *F*_diet(1,26)_ = 9.8, *p* < 0.01	*APOE3* control vs. *APOE4* control; *p* < 0.05 *APOE4* control vs. *APOE4* WD; *p* < 0.05
4E	*F*_APOE(1,27)_ = 4.7, *p* < 0.05	
4F	*F*_APOE(1,27)_ = 8.7, *p* < 0.01	*APOE3* WD vs. *APOE4* WD; *p* = 0.057
4G	*F* _diet(1,27)_ = 4.1, *p* = 0.053	
4H	*F* _diet(1,27)_ = 3.7, *p* = 0.064	

Abbreviations: apolipoprotein E genotype, *APOE*; Western diet, WD.

## Data Availability

The data presented in this study are available in the article.
